# Biological Markers and Alzheimer Disease: A Canadian Perspective

**DOI:** 10.4061/2010/978182

**Published:** 2010-08-08

**Authors:** Hyman M. Schipper

**Affiliations:** Department of Neurology and Neurosurgery, Centre for Neurotranslational Research, Lady Davis Institute for Medical Research, Jewish General Hospital, McGill University, 3755 Cote St. Catherine Rd. Montreal, QC, Canada H3T 1E2

## Abstract

Decreased *β*-amyloid_1-42_ and increased phospho-tau protein levels in the cerebrospinal fluid (CSF) are currently the most accurate chemical neurodiagnostics of sporadic Alzheimer disease (AD). A report (2007) of the Third Canadian Consensus Conference on the Diagnosis and Treatment of Dementia (2006) recommended that biological markers should *not* be currently requisitioned by primary care physicians in the routine investigation of subjects with memory complaints. Consideration for such testing should prompt patient referral to a specialist engaged in dementia evaluations or a Memory Clinic. The specialist should consider having CSF biomarkers (*β*-amyloid_1-42_ and phospho-tau) measured at a reputable facility in restricted cases presenting with atypical features and diagnostic confusion, but not as a routine procedure in all individuals with typical sporadic AD phenotypes. We submit that developments in the field of AD biomarker discovery since publication of the 3rd CCCDTD consensus data do not warrant revision of the 2007 recommendations.

## 1. Introduction

The advent of a biological marker that reliably indicates the presence of Alzheimer disease (AD) and distinguishes the latter from other dementing disorders would greatly assist the medical management of this common neurodegenerative condition. The successful integration of such a marker in routine clinical practice would confer the following benefits: (1) the accurate and expeditious diagnosis of sporadic AD, (2) curtailment of ancillary biochemical and imaging studies currently employed to exclude other causes of dementia, (3) the capacity to recognize AD in subjects with major affective disorders, clouded sensorium, depressed levels of consciousness, and other illnesses that often preclude assignment of a dementia diagnosis by conventional means, (4) possible surveillance of AD severity, progression, and impact of therapeutic interventions, (5) prognostication of conversion to incipient AD in individuals with mild cognitive impairment (MCI), and (6) treatment arm assignment and stratification of volunteers enrolled in clinical trials. In this paper, we review criteria for ideal biomarkers of sporadic AD, chemical biomarkers currently in vogue, and a national perspective on the clinical use of AD biomarkers in Canada based on the Third Canadian Consensus Conference on the Diagnosis and Treatment of Dementia [1].

## 2. Biological Markers and Sporadic AD

A biological marker of disease may be defined as a measurable change in the physical composition of an organism that indicates the presence of the illness. Biomarkers currently under investigation for the early diagnosis of AD include brain volume or activity measurements derived from neuroimaging techniques, such as positron emission tomography (PET) or magnetic resonance imaging (MRI) and chemical indices detected in various body fluids. Neuroimaging modalities are labor-intensive, expensive, and not universally available, prompting intense research efforts towards the development of effective chemical biomarkers and other practical neurodiagnostic tools. Chemical markers of AD fall within three general categories: (i) *genetic markers*, (ii) *genetic modifiers,* and (iii) *biological markers*. Mutant forms of amyloid precursor protein, presenilin-1, and presenilin-2 are proven *genetic markers *of AD. While useful for predicting disease in rare kindreds with familial AD (<10% of all AD cases), they play little or no role in tracking disease progression or efficacy of therapeutic intervention in these patients. Moreover, these genetic markers have little or no relevance for the management of individuals with the far more common, sporadic form of the disease [1, 2]. Carriers of the apolipoprotein E (*APOE*) *ε*4 allele, a *genetic modifier*, are at increased risk for the development of sporadic AD, manifest dementia symptoms earlier than *ε*4-negative persons with the disease, and exhibit accelerated conversion rates from MCI to AD [3]. However, testing for the *ε*4 allele cannot be used as a diagnostic marker of sporadic AD because its presence does not guarantee that that the disease exists or will occur nor does its absence exclude the condition. True *biological markers *of AD, in contradistinction to genetic markers and modifiers, inform on the presence or absence of AD at the time of measurement (“state” indicators) and may therefore serve as diagnostic modalities of the disease. 

## 3. Criteria for an “Ideal” Biological Marker of Sporadic AD

Principles set forth in a Consensus Report on Molecular and Biochemical Markers of AD sponsored by the Alzheimer's Association (US) and the National Institute on Aging have served as a guiding light for the development of AD biomarkers worldwide [2]. This landmark report recommended that an “ideal” biological marker of AD meet the following criteria [4]:

- reflect a fundamental aspect of CNS pathophysiology in AD (plausibility);
- indicate the actual presence of AD and not merely increased risk;
- exhibit high sensitivity and specificity (in the range of 80% or better for each);
- be efficacious in early or preclinical AD (e.g., MCI);
- monitor disease severity or rate of progression;
- indicate efficacy of therapeutic intervention;
- be noninvasive, inexpensive, and readily available.

In subsequent reports on this topic, it was also deemed desirable that (viii) the efficacy of the putative biomarker be corroborated by at least one other independent laboratory and that its accuracy (criterion (iii)) be demonstrated in discriminating AD not only from cognitively-healthy controls but from patients with various non-AD dementias [5].

## 4. Biological Markers of Sporadic AD

In this section, we review the utility of CSF *β*-amyloid_1–42_ (A*β*_1–42_) and tau/phospho-tau (p-tau) measurements as clinical biomarkers of sporadic AD. Other candidate chemical biomarkers of the disease currently commercially available or under investigation include urine AD7C-neuronal thread protein (marketed by Nymox Pharmaceutical Corp., Montreal), CSF and urinary F_2_-isoprostanes, other redox reporter molecules, plasma biospectroscopy, and a host of blood proteins, mRNAs, microRNAs, cholesterol metabolites, and transition metals. The latter will require further validation before they can be recommended for routine clinical use and will not be discussed further. Readers interested in these candidate biomarkers may consult recent literature from the author's laboratory and others on this topic [1, 6–19].

(i) *CSF *A*β*_1–42_: Amyloid fragments are plausible AD biomarkers because they represent a hallmark pathological process in the affected brain (senile plaque formation). Evidence from numerous studies worldwide indicates that concentrations of the amyloid peptide fragment A*β*_1–42_ are abnormally diminished in the CSF of patients with sporadic AD and MCI [19, 20]. A meta-analysis involving 18 studies of CSF A*β*_1–42_ as a diagnostic marker of AD revealed an effect size of 1.56 (95% CI: 1.43–1.69) [21]. In 2003, an analysis of CSF A*β*_1–42_ data derived from 13 studies (~600 AD and 450 control subjects), all utilizing the Innogenetics ELISA for the peptide, indicated an overall sensitivity and specificity of 80% and 90%, respectively, for distinguishing AD from cognitively-healthy controls [22]. However, CSF A*β*_1–42_ may decline in other degenerative CNS conditions including Lewy body dementia (LBD) [23–25], amyotrophic lateral sclerosis (ALS) [26], multisystem atrophy [27], and Creutzfeldt-Jakob disease (CJD) [24, 28]. Thus, in a multicentre study involving 150 AD, 100 normal elderly controls and 79 cases of non-AD dementia, the specificity of CSF A*β*_1–42_ in differentiating AD from normal subjects was 81% whereas it was only 59% relative to non-AD dementias [29]. Important data concerning the use of CSF biomarkers in the management of AD are now emerging from the Alzheimer Disease Neuroimaging Initiative (ADNI), a large, multi-institutional prospective study designed to correlate clinical phenotypes with imaging and chemical biomarkers in >800 rigorously-ascertained subjects with normal cognition, MCI, and AD [30]. A first such report [31] confirmed the stratification of cognitively normal, MCI and AD subjects based on declines in CSF A*β*_1–42_ levels (205.6 ± 55.1, 162.8 ± 56.0, and 143.0 ± 40.8 pg/ml for the 3 groups, respectively; *P* < .001). Moreover, baseline CSF A*β*_1–42_ concentrations successfully predicted the deterioration of neuropsychological measures in the normal and MCI cohorts (but not AD persons) over an ensuing 12-month period. *Plasma amyloid*: augmented plasma *β*-amyloid_1–42_ (A*β*_1–42_) concentrations have been reported in several kindreds with *familial* AD [32], but these families comprise a very small proportion of the entire AD population. Measurements of CSF or blood total A*β* peptide, A*β*_1-40_ or soluble APP*α*/*β* concentrations have thus far not proven useful in the diagnosis of sporadic AD [20, 22, 33–35] although identification of novel amyloid peptide fragments in AD biofluids using mass spectrometry techniques may still yield markers of diagnostic significance [19, 36].

(ii) *CSF total tau: *CSF total (t) tau reflects neurofibrillary tangle formation in the AD brain but is also a fairly nonspecific marker for neuronal destruction in a wide range of degenerative and nondegenerative CNS disorders. Elevated levels of total tau protein (t-tau) have been consistently encountered in AD CSF. An effect size of 1.31 (95% CI: 1.23–1.39) for CSF tau as an AD diagnostic was disclosed in a meta-analysis involving 35 studies [21]. In 2003, a review of CSF t-tau data from 41 studies (over 4000 AD and control subjects) that used either the Innogenetics or Athena ELISA disclosed a sensitivity and specificity for the diagnosis of AD of 80% and 90%, respectively (akin to the meta-analysis of CSF A*β*_1–42_) [22]. In the robust ADNI study [31], CSF t-tau increased progressively from 69.7 ± 30.4 to 101.4 ± 62.2 to 119.1 ± 59.6 pg/ml in normal, MCI, and AD subjects, respectively (*P* < .001). As in the case of CSF A*β*_1–42_, CSF t-tau is less effective in discriminating AD from other dementias, with specificities of 57% for suspected non-AD dementias [29] and 69% for autopsy-confirmed cases [24]. Elevated concentrations of CSF t-tau may also predict progression of cognitive deterioration in MCI, especially in patients without extensive periventricular white matter lesions [37]. High levels of CSF t-tau may also arise in fronto-temporal dementia (FTD) [38], vascular dementia [39], CJD, and (transiently) in acute ischemic stroke [40]. CSF t-tau values in LBD [23] and vascular dementia [41] may be intermediate between those of the cognitively-normal elderly and subjects with AD. Interestingly, 34% of individuals with FTD in one study exhibited significantly *suppressed* levels of CSF tau, a finding not seen in the AD cohort [42]. 

(iii) *CSF phospho-tau:* Phospho-tau isoforms are tenable AD biomarkers because they reflect a known pathophysiological process in AD brain (neurofibrillary tangle formation). A number of laboratories have documented significant increases in levels of hyperphosphorylated tau in AD CSF relative to cognitively-intact controls using antibodies against various phosphorylated epitopes of tau (p-tau). CSF p-tau is elevated in “incipient AD” [43] and MCI [44, 45] and is therefore a relatively early biomarker of the disease. In the aforecited ADNI report [31], levels of threonine 181 p-tau in the CSF of persons with normal cognition, MCI, and AD were, respectively, 24.9 ± 14.6, 35.5 ± 18.0, and 41.6 ± 19.8 pg/ml (*P* < .001). Use of CSF p-tau to monitor disease progression may be limited by dilutional factors unless combined with MRI measurements of hippocampal atrophy [46]. Of note, p-tau levels in AD CSF are reportedly elevated relative to other dementing and nondementing neurological disorders [22, 47, 48]. As such, and in contradistinction to t-tau, enhanced CSF p-tau levels may differentiate AD from FTD [49, 50], Lewy body dementia [51], vascular dementia [52], PD [53], ALS, acute stroke [54], schizophrenia [55], and major depression [53]. Despite a previous report to the contrary [56], CSF concentrations of threonine 181 p-tau may be augmented in sporadic and variant CJD [57]. 

(iv) *CSF *A*β*_1–42_ *and p-tau combined: *CSF A*β*_1–42_ and p-tau, when measured together, exhibit sensitivities and specificities (versus other dementing disorders) in the range of 80%–90% [58]. The positive and negative predictive values of the combined test are 90% and 95%, respectively, assuming a prevalence rate of 45% [20]. This biomarker combination reflects disease pathophysiology (*vide supra*), identifies AD in early stages (e.g., MCI), and is relatively inexpensive. Some posit that CSF A*β*_1–42_ represents the *stage* of AD (with concentrations diminishing progressively as a function of disease duration), while t-tau and p-tau are indicators of disease *intensity* (with higher CSF levels connoting more rapid progression) [59]. It has been suggested that the extent of CSF tau elevation and A*β*_1–42_ suppression may correlate with the *APOE* *ε*4 allele burden [60] although the extent to which genetic factors impact CSF biomarker levels remains uncertain. In patients with MCI, the biomarker combination may prognosticate for imminent conversion to AD with sensitivities/specificities in the range of 83%–90% [61, 62]. The markers also exhibited efficacy in delineating “nonprogressors” in “mixed” (amnestic and nonamnestic) MCI over a 3-year median follow-up period [63] and may assist in distinguishing MCI from anxiety and depression [64]. A large European-American multi-institutional trial employed a cutoff CSF A*β*_1–42_/p-tau ratio predetermined from an established AD cohort (at 85% sensitivity) to detect AD in 750 MCI individuals followed longitudinally for at least two years or until dementia intervened. The investigators identified incipient AD in the MCI subjects with 83% sensitivity, 72% specificity, 62% positive predictive value, and 88% negative predictive value. The authors concluded that although the test was accurate in identifying incipient AD, intersite assay variability limited its performance relative to previous results from single-centre studies, underscoring the need for standardization of clinical procedures and analytical techniques [65]. In another recent multicentre study, AD-like CSF biomarker ratios were noted to be more frequent among individuals with subjective (but no objective) cognitive impairment (SCI; 52%) than in healthy controls (31%; *P* < .01), suggesting that AD may be the cause of SCI (and not only MCI) in a significant proportion of elderly subjects [66]. To our knowledge, CSF A*β*_1–42_ and tau determinations have not yet proven helpful as indices of therapeutic efficacy in AD. 

(v)* CSF biomarkers: further considerations:* (a) In the majority of AD biomarker studies, the validity of the data were limited because receiver operating characteristic curves (plotting the relationship between sensitivities and specificities) were generated on the basis of clinical diagnoses without autopsy corroboration. While *prospective* AD biomarker studies are in principle more valuable than retrospective analyses, the former are less likely to include neuropathological diagnoses [67]. (b) The immunoassay procedures invoked to measure CSF A*β*_1–42_ and tau are not trivial, and interlaboratory variability is commonplace. (c) Athena Neurosciences charges US$905 to MDs and $1,335 to insurance companies for the combined tau and CSF A*β*_1–42_ assays per sample. It was announced this year that the cost of AD biomarkers would be defrayed by the Canadian government pending documentation of need. Regardless, the cost may not be prohibitive if it obviates the need for additional testing (e.g., neuroimaging). (d) In a study of 342 AD, MCI, and cognitively normal individuals subjected to 428 research lumbar punctures, the adverse effect rate was low (e.g., post-LP headaches in 0.93%), and the procedure was generally well tolerated (low pain and anxiety scores in visual analog scales) [68]. Yet, CSF examination by lumbar puncture is more invasive than venipuncture or urine analysis and currently not suitable for mass screening of elderly persons with AD risk factors or mild memory impairment. The latter could warrant revisiting in the event that effective measures to prevent AD were to become available. 

## 5. The Third Canadian Consensus Conference on the Diagnosis and Treatment of Dementia

Canadian Consensus Conferences on the Diagnosis and Treatment of Dementia were held in 1989, 1998 and, most recently, in March 2006 (Montreal) in attempt to standardize the diagnostic and therapeutic management of AD and related dementias in our country [69]. The structure and organization of the 3rd CCCDTD followed guidelines of the AGREE collaboration [70]. The project was funded by major government health institutes, geriatric and Alzheimer societies, and unrestricted grants from the pharmaceutical industry. Acknowledged leaders representing the disciplines of neurology, geriatric medicine, geriatric psychiatry, and neuropsychology, with liaisons from family practice, participated in the 3rd CCCDTD. PubMed and Embase electronic databases (supplemented by individual investigator files) spanning from January 1996 to December 2005 were surveyed for pertinent literature on nine designated topics. Publications were included for review based on their quality as determined by Jadad criteria [71]. The strength of evidence was graded according to the Canadian Task Force on Preventive Health Care [72]: 

(I) Evidence obtained from at least one properly randomized controlled trial. (II-1) Evidence obtained from well-designed controlled trials without randomization, (II-2) Evidence obtained from well-designed cohort or case-control analytic studies preferably from more than one centre or research group, or (II-3) evidence obtained from comparisons between times or places with or without the intervention. Dramatic results in uncontrolled experiments are included in this category. (III) Opinions of respected authorities based on clinical experience, descriptive studies, or reports of expert committees. The valence and strength of recommendations were assigned using the following grading system [73, 74]. (A) There is good evidence to support this maneuver. (B) There is fair evidence to support this maneuver. (C) There is insufficient evidence to recommend for or against this maneuver, but recommendations might be made on other grounds. (D) There is a fair evidence to recommend against this procedure. (E) There is good evidence to recommend against this procedure. Background papers and sets of recommendations for each topic were posted online and voted upon by all conferees. Recommendations receiving at least 80% support were considered to have achieved consensus. The full list of approved recommendations is available on the websites of the 3rd CCCDTD (http://www.cccdtd.ca/) and the Alzheimer Society of Canada (http://www.alzheimer.ca/). Eighteen background articles accruing from this exercise were published in the October 2007 issue of *Alzheimer's & Dementia*.

## 6. 3rd CCCDTD: Role of Biomarkers

To ascertain the role of biomarkers in AD for the 3rd CCCDTD, the author reviewed a total of 186 papers: 137 generated from surveillance of the electronic literature (see Section 5) using the search terms “Alzheimer disease” AND (“Biological Marker” OR “Biomarker”), and an additional 49 articles from the author's files. The analysis led to the following *conclusions * [1]:

(i) AD is a public health concern of epidemic proportions for which current diagnostic (and therapeutic) modalities remain insufficient. 

(ii) The advent of a biological marker that differentiates early, sporadic AD from normal aging and other dementing disorders would represent a significant advance in the evaluation and management of this neurodegenerative disorder. An accurate, minimally invasive biological marker of early sporadic AD would serve the public interest by facilitating patient and family counselling, enabling stratification of subgroups for enrolment in clinical drug trials, and improving the interpretation of treatment outcomes. The introduction of a chemical marker that differentiates “malignant” MCI cases at high risk for deterioration to AD from neuropsychologically similar cases destined to manifest “benign” aging-associated memory changes would be particularly useful. Biomarkers may also prove helpful in situations where concomitant medical or psychiatric conditions confound or preclude neuropsychological testing, for example, major depression, delirium, suppressed consciousness, or individuals who are otherwise uncooperative for detailed cognitive testing. (Although conjectural and not listed among the published conclusions of the 3rd CCCDTD, it should prove interesting to determine whether measurement of AD biomarkers in patients with normal pressure hydrocephalus assists in the selection of appropriate candidates for (and improves the success rate of) surgical shunting.)

(iii) Although several candidate biomarkers of sporadic AD have been identified and commercialized, none currently fulfills criteria for an ideal test (see Section 3).

(iv) Decreased A*β*_1–42_ and increased phospho-tau protein concentrations in the CSF are currently the most accurate and reproducible chemical neurodiagnostics of sporadic AD. These biomarkers also show promise as prognosticators in subjects with MCI. However, CSF evaluation by spinal tap remains impractical for mass screening of elderly individuals with symptoms of memory impairment or AD risk factors. 

(v) Platelet APP isoform ratios, plasma or urinary F_2_-isoprostane levels, blood biospectroscopy, and other modalities under investigation may fulfil several criteria for an “ideal” biological marker of early sporadic AD (Section 3). However, further experimentation and validation will be needed before these candidate biomarkers can be considered for clinical use. Similarly, all AD biomarker candidates arising from mass spectrometry and other proteomic applications [19, 75, 76] will require stringent clinical evaluation for their suitability as bonafide diagnostic tools.

(vi) Given the complexity of AD pathology, it is likely that combinations of individual biomarkers will provide more accurate diagnostic and prognostic data that any single marker assayed in isolation (akin to use of multiple biochemical indices to characterize liver failure, cardiac ischemia, or connective tissue disease).

On the basis of the literature analysis and aforementioned conclusions, the following *recommendations* reached consensus (see Section 5) and were published by the 3rd CCCDTD [1].

### 6.1. To Primary Care Physicians

 (i) “Biological markers for the diagnosis of AD should not, at this juncture, be included in the battery of tests routinely used by primary care physicians to evaluate subjects with memory loss (Grade C, Level 3). Consideration for such specialized testing in an individual case should prompt referral of the patient to a specialist engaged in dementia evaluations or a Memory Clinic.”

### 6.2. To Specialists

 (i) “Although highly desirable, there currently exist no blood- or urine-based AD diagnostics that can be unequivocally endorsed for the routine evaluation of memory loss in the elderly (Grade C, Level 3). The non-invasiveness of such tests, if and when they become available, would be suitable for mass screening of subjects with memory loss presenting to specialists in their offices and Memory Clinics.

(ii) Due to their relative invasiveness and availability of other fairly accurate diagnostic modalities (clinical, neuropsychological and neuroimaging), CSF biomarkers should not be routinely performed in all subjects undergoing evaluation for memory loss (Grade D, Level 2).

(iii) CSF biomarkers may be considered in cases where there are atypical features and diagnostic confusion. CSF biomarkers may be useful in differentiating frontal variants of AD from FTD (Grade B, Level 2).

(iv) When a decision to obtain CSF biomarkers is made, combined A*β*_1–42_ and p-tau concentrations should be measured by validated ELISA (Grade A, Level 1). It may be best to convey the CSF samples to a centralized facility (commercial or academic) with a track record in generating high-quality, reproducible data.

(v) CSF biomarker data in isolation are insufficient to diagnose or exclude AD (Grade C, Level 3). They should be interpreted in light of clinical, neuropsychological, other laboratory and neuroimaging data available for the individual under investigation.”

It is the opinion of the author and Dr. Howard Chertkow (Chair, 3rd CCCDTD, personal communication) that developments reported in the field of AD biomarker discovery since publication of the 3rd CCCDTD consensus data do not warrant revisal of the 2007 recommendations. However, this remains an area of intensive research worldwide and further insights from large-scale initiatives such as ADNI, or validation of blood- or urine-based markers of the disease, may prompt a sea-change in the way AD biomarkers are exploited in Canadian clinics.

##  Disclosures 

Hyman M. Schipper has served as consultant to Osta Biotechnologies, Molecular Biometrics Inc., TEVA Neurosciences and Caprion Pharmaceuticals. He holds stock options in Osta and equity in Molecular Biometrics.
